# Optimization of sentinel lymph node biopsy in breast cancer using an operative gamma camera

**DOI:** 10.1186/1477-7819-5-132

**Published:** 2007-11-17

**Authors:** Carole Mathelin, Samuel Salvador, Sabrina Croce, Norosoa Andriamisandratsoa, Daniel Huss, Jean-Louis Guyonnet

**Affiliations:** 1 Service de Gynécologie-Obstétrique, Hôpital Civil, 1 place de l'Hôpital. F-67091 Strasbourg Cedex, France; 2 Institut Pluridisciplinaire Hubert Curien. UMR 7178 – CNRS/IN2P3 et ULP. BP 28 – 23 rue du Loess. F-67037 Strasbourg Cedex 02, France; 3 Département d'anatomie-pathologique. Hôpital de Hautepierre, Avenue Molière. F-67200 Strasbourg Cedex, France; 4 Service de Médecine Nucléaire. Hôpital Civil, 1 place de l'Hôpital. F-67091 Strasbourg Cedex, France

## Abstract

**Background:**

Sentinel lymph node (SLN) procedure is now a widely accepted method of LN staging in selected invasive breast cancers (unifocal, size ≤ 2 cm, clinically N0, without previous treatment). Complete axillary clearance is no longer needed if the SLN is negative. However, the oncological safety of this procedure remains to be addressed in randomized clinical trials. One main pitfall is the failure to visualize SLN, resulting in incorrect tumor staging, leading to suboptimal treatment or axillary recurrence. Operative gamma cameras have therefore been developed to optimize the SLN visualization and the quality control of surgery.

**Case presentation:**

A 44-year-old female patient with a 14-mm infiltrative ductal carcinoma underwent the SLN procedure. An operative gamma camera was used during and after the surgery. The conventional lymphoscintigraphy showed only one SLN, which was also detected by the operative gamma camera, then removed and measured (9.6 kBq). It was analyzed by frozen sections, showing no cancer cells. During this analysis, the exploration of the axillary area with the operative gamma camera enabled the identification of a second SLN with low activity (0.5 kBq) that conventional lymphoscintigraphy, surgical probe and blue staining had failed to visualize. Histological examination revealed a macrometastasis. Axillary clearance was then performed, followed by a postoperative image proving that no SLN remained. Therefore, the use of the operative gamma camera prevented an under-estimation of staging which would have resulted in a suboptimal treatment for this patient.

**Conclusion:**

This case report illustrates that an efficient operative gamma camera may be able to decrease the risk of false negative rate of the SLN procedure, and could be an additional tool to control the quality of the surgery.

**Trial Registration:**

ClinicalTrials.gov Identifier: NCT00357487

## Background

As lymph node (LN) metastasis is one of the most important prognostic factors for survival, the assessment of regional LN is essential in the staging of breast cancer, ascertaining a prognosis, and determining of optimal adjuvant treatments. The sentinel node (SLN) procedure consists of recognizing and removing the first LN that filter lymphatic fluid from the tumor. It is now a widely accepted method of LN staging in selected invasive breast cancer (unifocal, size ≤ 2 cm, clinically N0, without previous treatment). The complete axillary clearance is no longer needed if the SLN is normal [1]. However, its oncological safety remains to be addressed in randomized clinical trials [2]. One main pitfall is the failure to visualize SLN, resulting in incorrect tumor staging, leading to suboptimal treatment or axillary recurrence. Several factors including advanced age, high body mass index, massive metastasis invasion [3] or tumor location other than upper outer quadrant [4] may contribute to SLN non-visualization.

Operative gamma cameras (OGC) have therefore been developed to optimize the SLN visualization and the quality control of surgery [5-10]. In our ongoing clinical trial [11], an OGC, named CarolIReS, with a current field of view (FOV) of 50 × 50 mm^2 ^is being used [12]. It is made of a lead parallel collimator, a thin Gd_2_SiO_5_:Ce scintillating crystal and a multianode photomultiplier (10 mm spatial resolution and 2.2 cps.kBq^-1 ^sensitivity for the ^99m^Tc). We present the first case in which OGC, used at the end of the surgery, enabled the detection of a residual metastatic SLN.

## Case presentation

**Figure 1:**
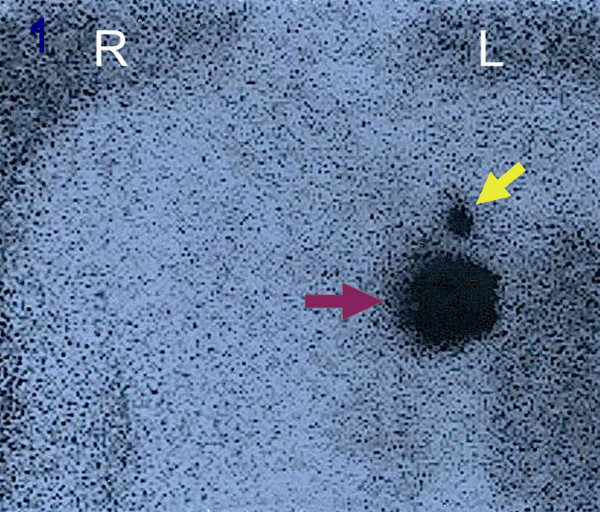
The anterior view of the conventional lymphoscintigraphy shows one SLN indicated by the yellow arrow. The purple arrow indicates the injection site.

**Figure 2:**
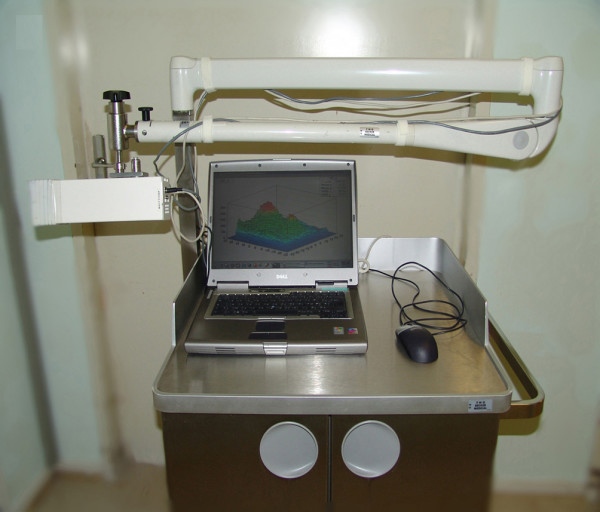
Picture of the CarolIReS gamma camera built at the IPHC, Strasbourg, France.

**Figure 3:**
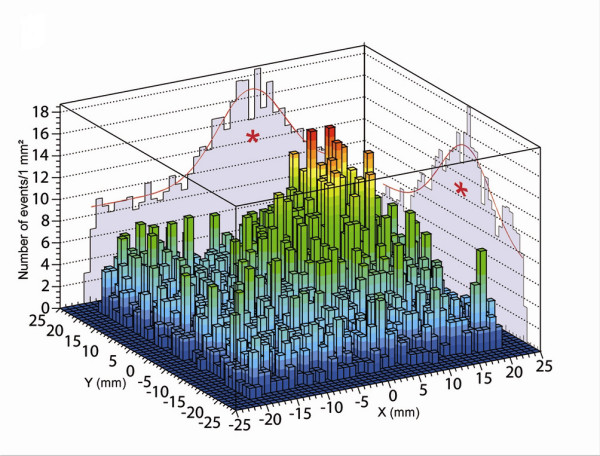
Preoperative image of the axillary area obtained with the CarolIReS gamma camera. The x and y cumulative profiles (red stars) are used to determine the planar coordinates and the anatomical depth of the first SLN.

A 44-year-old female patient with a 14 mm infiltrative ductal carcinoma, Scarf-Bloom-Richardson grade 2 (diagnosed by core biopsy) was enrolled in the clinical study [11] after informed consent had been obtained. She was free of chemotherapy, loco-regional radiotherapy, prevalent axillary LN and her body mass index was normal (21 kg/m^2^). The tumor was located in the upper outer quadrant of the left breast. The SLN procedure was initiated the day prior to the surgery using 4 subareolar injections of rhenium sulfur colloidal and technetium (^99m^Tc) (0.4 ml, 22 MBq (595 μCi), Cis bio international^®^, France). Scintigraphic images (anterior, lateral and oblique views), obtained 3 hours after rhenium sulfur injections with a conventional gamma camera (Hélix^®^, Elscint, Haïfa), showed one SLN (figure 1). Immediately before surgery, when the patient was in operative position, four images (2 minutes data acquisition each) were obtained with the OGC to map the full axillary area (figure 2 represents a picture of the OGC). The precise localization of this SLN and an estimation of its depth were determined from the data by using the width of the image cumulative profiles (figure 3).

**Figure 4:**
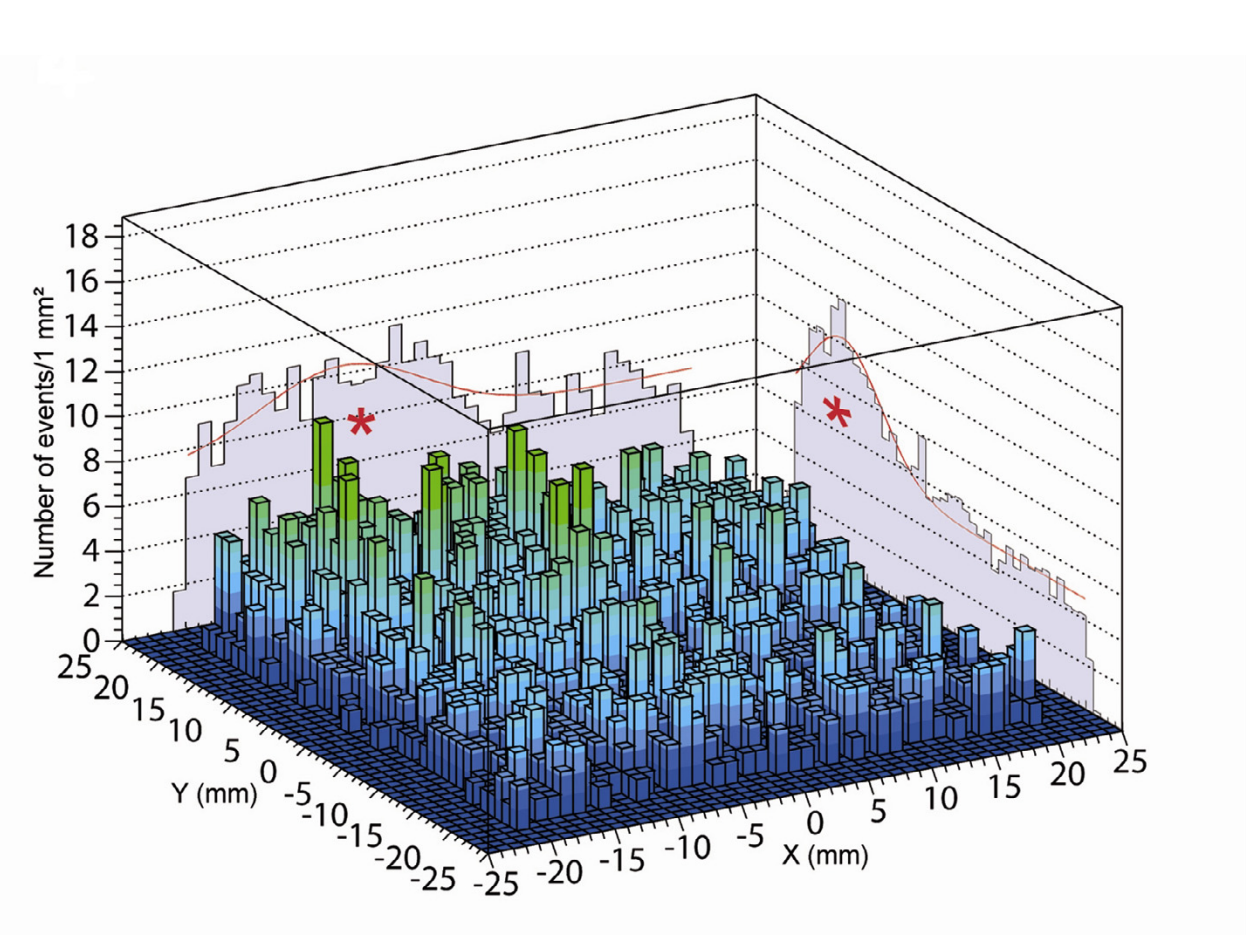
Peroperative image of the axillary area obtained with the CarolIReS gamma camera. The x and y cumulative profiles (red stars) are used to determine the planar coordinates and the anatomical depth of the second SLN.

The surgery began with 4 subareolar injections of Methylene Blue (Lab Aguettant^®^, Lyon, France), and the search for the SLN was carried out using the CarolIReS intra-operative probe [13], which detected only one SLN. This SLN was blue stained and its measured activity was 9.6 kBq (0.26 μCi). It was subsequently submitted for frozen section analysis, which showed the absence of cancer cells. During this analysis, the axillary area was thoroughly palpated by the surgeon and no nodes showing signs of extensive tumor involvement were identified. A second exploration of this area was then performed using the OGC enabling the identification of a second SLN located at a distance from the first one of less than the spatial resolution of the OGC (figure 4). This SLN was not stained and had a low activity of 0.5 kBq (0.01 μCi) concentrated in its hilum.

**Figure 5:**
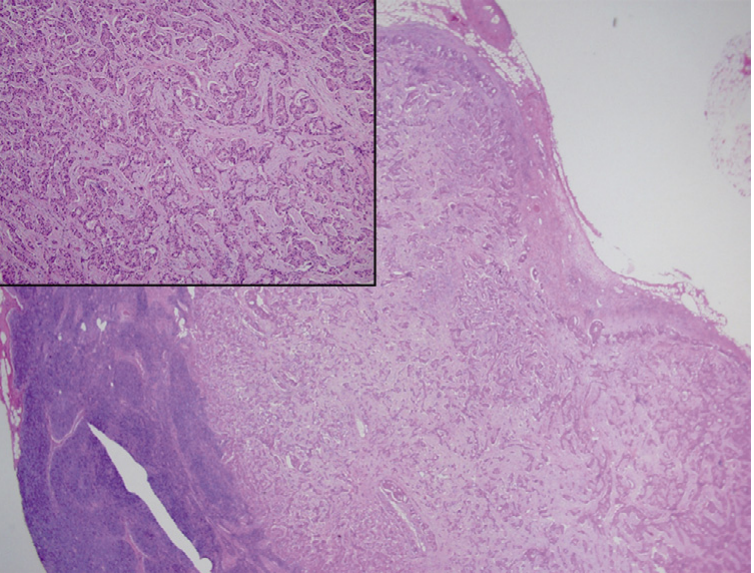
Ductal breast carcinoma metastasis destroying nodal architecture (Hematoxilin and Eosin ×20). Inset: metastatic cells (Hematoxilin and Eosin ×200) in the second SLN.

**Figure 6:**
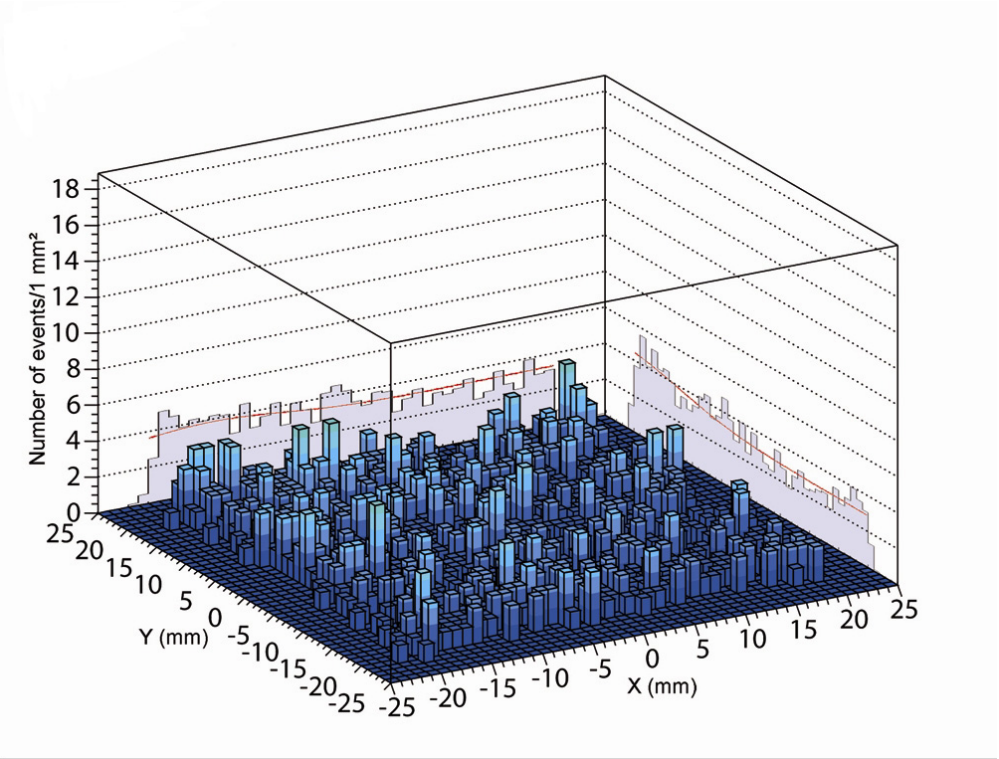
Postoperative image of the axillary area obtained with the CarolIReS gamma camera.

The histological examination revealed an 8-mm macrometastasis occupying the whole structure of this SLN without residual sinusoidal macrophages (figure 5). Axillary clearance was then performed and each of the 11 LN removed was negative. Axillary surgery was followed by lumpectomy. A postoperative OGC image proved that no SLN remained (figure 6) and was considered a quality control document.

## Discussion

This is the first published case in which an OGC enabled the detection of a non-stained metastatic SLN, which was neither diagnosed by conventional lymphoscintigraphy, nor by operative probe, nor by palpation of the axillary area performed by an experienced surgeon. This can be partially explained by the very low activity of this SLN (0.5 kBq). It corresponds to 0.02‰ of the injected dose, which is inferior to our mean measured value of 0.5‰. Metastatic SLN sometimes have very low activity, because they contain numerous cancer cells and few macrophages, the latter being the cells that retain radiocolloïdes [14]. In our experience, the activity of metastatic SLN is less than 10 kBq in more than half of the cases. So the detection efficiency of OGC must be good, and that of the one we used in this reported case was 2.2 cps.kBq^-1^. Future clinical trials will have to determine whether or not there is an inferior threshold for the activity of the SLN which should be removed.

In addition, ongoing trials using OGC will have to evaluate if the latter lead to unnecessary SLN removal and increase morbidity or if they decrease the false negative rate (FNR). However, it has been demonstrated that the identification of multiple SLN, when present, reduced the FNR. For example, Goyal *et al*. [15] studied a series of 803 patients with breast cancer who underwent successful SLN procedure followed by an axillary clearance. In patients who had one SLN harvested, the FNR was 10%, compared with 1% in patients who had 3 or more SLN removed. Factors associated with finding multiple SLN (62.4% of patients) were age less than 50 years, low body mass index, tumor in the outer half of the breast, SLN visualization on lymphoscintigraphy and an interval of 12 hours or less between radioisotope injection and SLN procedure. For 99.6% of node-positive tumors, metastases were detected within the first 4 SLN removed and the authors suggested that the removal of more than 4 SLN was unnecessary [15]. Ongoing clinical trials will have to discuss precisely the usefulness of OGC according to the number of SLN found with conventional procedures (less than or equal to 4 or greater than 4).

## Conclusion

This case report illustrates that an efficient OGC may be able to decrease the risk of FNR of the SLN procedure, and could be an additional tool to control the quality of the surgery. But a precise evaluation of OGC should be based upon data from a large number of patients. Our ongoing clinical trial [11] will be soon published and a new clinical trial, including 100 breast cancer patients, will begin at the end of 2007 to evaluate the usefulness of the new CarolIRes OGC (FOV : 100 × 100 mm^2^) in the SLN procedure.

## Abbreviations

FNR: false negative rate

FOV: field of view

OGC: operative gamma camera

SLN: sentinel lymph node

## Competing interests

The author(s) declare that they have no competing interests.

## Authors' contributions

**CM **carried out the clinical trial, performed the surgical treatment and drafted the manuscript. **SC **performed the pathological analysis and helped to draft the manuscript. **NA **performed the lymphoscintigraphy and was medical investigator of the clinical trial. **JLG**, **SS **and **DH **were scientific investigators of the clinical trial, helped to draft the manuscript and performed the statistical analysis of the clinical trial. All authors read and approved the final manuscript.
